# Effect of Vitamin D_3_ on Chemerin and Adiponectin Levels in Uterus of Polycystic Ovary Syndrome Rats

**DOI:** 10.3390/cells12162026

**Published:** 2023-08-08

**Authors:** Karolina Pich, Jesika Rajewska, Kinga Kamińska, Marcelina Tchurzyk, Agata Szlaga, Patryk Sambak, Anna Błasiak, Małgorzata Grzesiak, Agnieszka Rak

**Affiliations:** 1 Laboratory of Physiology and Toxicology of Reproduction, Institute of Zoology and Biomedical Research, Jagiellonian University, 30-387 Krakow, Poland; karolina.pich@doctoral.uj.edu.pl (K.P.); jesika.rajewska@student.uj.edu.pl (J.R.); 2 Doctoral School of Exact and Natural Sciences, Jagiellonian University, 30-348 Kraków, Poland; kinga.kaminska@doctoral.uj.edu.pl (K.K.); patryk.sambak@doctoral.uj.edu.pl (P.S.); 3 Department of Endocrinology, Institute of Zoology and Biomedical Research, Jagiellonian University, 30-387 Krakow, Poland; marcelina.tchurzyk@student.uj.edu.pl (M.T.); m.e.grzesiak@uj.edu.pl (M.G.); 4 Department of Neurophysiology and Chronobiology, Institute of Zoology and Biomedical Research, Jagiellonian University, 30-387 Krakow, Poland; agata.szlaga@doctoral.uj.edu.pl (A.S.); anna.blasiak@uj.edu.pl (A.B.)

**Keywords:** vitamin D_3_, chemerin, adiponectin, PCOS, uterus, rats

## Abstract

Background: Polycystic ovary syndrome (PCOS) is an endocrine disorder with disrupted uterus structure and function. A positive effect of vitamin D_3_ (VD_3_) in female reproduction was observed. Chemerin (RARRES2) and adiponectin (ADIPOQ) are the main adipokines whose levels are altered in PCOS patients. Therefore, the aim of this study was to investigate the impact of VD_3_ supplementation on RARRES2 and ADIPOQ levels in the uterus of PCOS rats. Methods: We analyzed the plasma levels and uterine transcript and protein expression of RARRES2 and ADIPOQ and their receptors (CCRL2, CMKLR1, GPR1, and ADIPOR1 and ADIPOR2, respectively) in rats with letrozole-induced PCOS. Results: In control animals, VD_3_ did not change plasma levels of both adipokines, while in PCOS rats supplemented with VD_3_, they returned to control levels. The expression of RARRES2 and all investigated receptors increased in the uterus of VD_3_-treated rats; however, the levels of *Rarres2* and *Gpr1* genes remained unchanged. VD_3_ supplementation decreased RARRES2, CMKLR1, and GPR1 but increased CCRL2 level to the control value. In the uterus of VD_3_-treated rats, the transcript and protein levels of ADIPOQ and both receptors ADIPOR1 increased. At the same time, VD_3_ supplementation induced an increase in *Adipoq*, *Adipor1*, and *Adipor2* gene expression and restored protein levels to control level values. Conclusions: our findings indicate a new mechanism of VD_3_ action in the uterine physiology of PCOS rats.

## 1. Introduction

Polycystic ovary syndrome (PCOS) is an endocrine–metabolic disorder commonly affecting 20–25% of women of reproductive age [1]. The diagnosis is particularly based on two of three criteria: oligo- or anovulation, clinical/biochemical hyperandrogenism, and polycystic ovarian morphology. Women affected with PCOS display hormonal imbalances such as elevated circulating luteinizing hormone (LH), testosterone (T), progesterone (P_4_), sex hormone-binding globulin (SHBG), and anti-mullerian hormone (AMH) [2]. Moreover, PCOS is associated with metabolic problems, especially insulin resistance, hyperinsulinemia, obesity, impaired glucose tolerance, and type 2 diabetes mellitus [1]. Additionally, literature data suggest that women with PCOS, independent of ovulatory status, have reduced reproductive potential [3] associated with abnormalities in oocyte morphology as well as lower rates of oocyte maturation and fertilization [4]. Moreover, the latest literature data indicate that women with PCOS exhibit pregnancy compilations like a three-to-fourfold increased risk of pregnancy-induced hypertension and preeclampsia (PE), a threefold increased risk of gestational diabetes, and a twofold higher chance for premature delivery [5]. In this regard, a “uterus factor” has been suggested to play a crucial role in infertility and poor pregnancy outcomes in PCOS women. The endometrial function of women with PCOS is altered including abnormalities in thickness, cell cycle, and apoptosis, as well as a disturbance in the regulation of enzymatic and metabolic pathways [6]. For example, in the endometrium tissue of PCOS women, the protein expression of B-cell lymphoma 2 was downregulated; on the other hand, the gene Fas cell surface death receptor was upregulated [7]. Endometrial alterations in PCOS women also include the enhanced expression of receptors for estrogen (ERα), P_4_ (PRα), and androgen (AR), and the coactivators of AR and ERα [6]. Moreover, it has been shown that the endometrial immune environment in PCOS is altered by increased levels of tumor necrosis factor and nuclear factor kappa B [8]. Interestingly, women with PCOS have a significantly higher risk of endometrial cancer [9].

Recently, the gene and protein expression of VD_3_, its receptor (VDR), and metabolic enzymes (CYP27B1 and CYP24A1) have been reported in the endometrium and myometrium [10]. Disrupted VD_3_ levels and metabolism were described in reproductive pathologies such as PCOS, endometriosis, and uterine leiomyoma [11,12]. The latest data demonstrated reduced calcidiol levels in PCOS women, suggesting a relationship between VD_3_ deficiency and the occurrence of many PCOS symptoms [13]. VD_3_ deficiency is often associated with disturbed calcium metabolism, which in women with PCOS may inhibit follicle maturation and ovulation. Moreover, a deficiency of plasma level of VD_3_ in PCOS women [14] also reduces the activity and expression of aromatase, which disturbs the conversion of androgens to estrogens. Recent studies have shown that in PCOS rats, the supplementation of VD_3_ reduced the thickness of the endometrium and decreased the immunohistochemical staining of caspase-3 and proliferative marker Ki67 as well as the serum AMH level [15,16]. In addition, the supplementation of VD_3_ affects the plasma chemerin level (RARRES2, retinoic acid 109 receptor responder protein 2) in a rat model of PE [17], and adiponectin (ADIPOQ) levels in diabetic [18] as well as PCOS [19] patients. However, the possible effects of VD_3_ on uterine adipokines level in PCOS have not been studied. Therefore, in the present study, we examined the impact of VD_3_ supplementation on the transcript and protein levels of RARRES2 and ADIPOQ, as well as their receptors in the uterus and plasma of PCOS rats.

It is a well-known fact that adipose tissue plays a crucial role in the development and progression of PCOS, manifested by secreting bioactive adipokines, which can modulate metabolism and reproduction [20]. Recently, there has been a growing interest in findings of the changes in circulating levels of adipokines in women with PCOS, and such meta-analysis studies demonstrated higher plasma levels of RARRES2 and a decreased concentration of ADIPOQ in PCOS patients compared to the control subjects [21]. Both RARRES2 and ADIPOQ are adipokines that are mainly produced by adipose tissue and regulate food intake, energy homeostasis, as well as insulin sensitivity by acting via the receptors C-C motif chemokine receptor-like 2 (CCRL2), chemerin chemokine-like receptor 1 (CMKLR1), and G protein-coupled receptor 1 (GPR1) (for RARRES2), and via adiponectin receptor-1 (ADIPOR1) and -2 (ADIPOR2) (for ADIPOQ) [20]. Previous studies demonstrated the expression of RARRES2 and ADIPOQ as well as their receptors in the endometrium in pigs [22,23] and humans [24] as well as ADIPORs in the uterus of pregnant rats [25]. Moreover, both adipokines regulate endometrium physiology by controlling the expression of genes, which are involved in cell proliferation, programmed cell death, angiogenesis, inflammation, and endocrinology [26,27].

The advantages of using the rat model of PCOS include the ability to provide a controlled environment, as well as capitalizing on the short life cycle to explore both reproductive and long-term metabolic effects [28]. Moreover, letrozole-induced PCOS model rats exhibit acyclicity and cystic ovarian morphology corresponding to human PCOS, as well as elevated serum LH levels [29]. It is also important to be aware of limitations, such as that rodents are multiovulatory species with some polymorphic variations of genes coding steroidogenic enzymes [30], which may have an impact on the outcome [31]. Additionally, limitations may also vary between the rat and female uterus. Humans have lightbulb-shaped uteri with mixed muscle layers, whereas rats have two-horned uteri with defined muscle layers [32]. The latest data showed that adenosine triphosphate-sensitive potassium channels may play different roles in the rat and human myometrium [32].

We hypothesize that the supplementation of VD_3_ in PCOS rats affects the transcript and protein levels of RARRES2 and ADIPOQ, as well as its receptors in the uterus and plasma levels of both adipokines. Therefore, the aim of the current study was to (i) confirm the induction of PCOS in rats by measuring plasma levels of steroids: T and estradiol (E_2_) and metabolic parameters: glucose, triglycerides, total cholesterol, and high-density lipoprotein (HDL) levels as well as the characterization of ovarian and uterus histology; (ii) describe plasma levels of RARRES2 and ADIPOQ; and (iii) mRNA and protein expression of RARRES2 and its receptors, CCRL2, CMKLR1, GPR1, as well as ADIPOQ, and its receptors ADIPOR1 and ADIPOR2 in the uterine of control and PCOS-induced rats.

## 2. Materials and Methods

### 2.1. Reagents

TRI reagent solution (cat. no. 15596018), a high-capacity cDNA reverse transcription kit (cat. no. 4368814), and tissue protein extraction reagent (cat. no. 78510) were purchased from ThermoFisher Scientific, Waltham, MA, USA. In total, 4–20% mini protean-gels and trans blot turbo mini PVDF (cat. no. 1704156) were purchased from Bio-Rad Laboratories, Hercules, CA, USA. WesternBright Sirius HRP substrate (cat. no. K-12043 D30) was purchased from Advansta Inc., San Jose, CA, USA. Letrozole (cat. no. L6545), 3′3′-aminopropyl-triethoxysaline-coated (cat. no. A-3648), eosin Y (cat. no. 1.09844), dibutylphtalate xylene (cat. no. 06522), and 3,3′-diaminobenzidine (cat. no. 857734) were purchased from Sigma-Aldrich, St. Louis, MO, USA. Hematoxylin QS (cat. no. H-3404) and StreptABCcomplex-HRP (cat. no. PK-6100) were purchased from Vector Laboratories, Newark, CA, USA.

### 2.2. Animals and Experimental Protocol

Six-week-old female Wistar rats (weighing 176.9 g ± 14.54 g) were purchased from the Faculty of Pharmacy JU Medical College (Kraków, Poland). Animals were housed in controlled conditions of temperature, humidity, and light (LD 12/12) with ad libitum availability of food and water. Rats were allowed to acclimate for one week before subsequent one-week-long handling and treatment. All experimental protocols listed herein were approved by the Local Animal Ethics Committee, Poland (permit no. 70/2021, Krakow, Poland).

**Figure 1:**
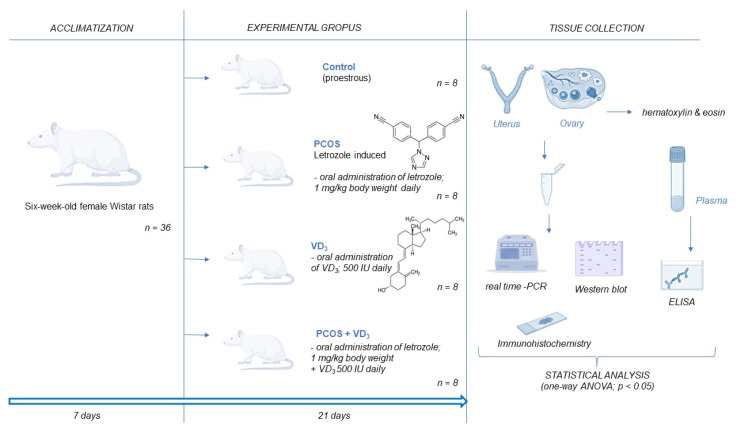
Experimental protocol. ANOVA, analysis of variance. ELISA, enzyme-linked immunosorbent assay. PCOS, polycystic ovarian syndrome. PCR, polymerase chain reaction. VD_3_, vitamin D_3_.

PCOS was induced in rats via the oral administration of a nonsteroidal aromatase inhibitor letrozole [33]. Briefly, rats (*n* = 32) were randomly assigned to one of the four experimental groups: control (in proestrous), VD_3_ (500 IU VD_3_/day, Vigantol 20,000 IU/mL, P&G Health, Darmstadt, Germany)-treated, PCOS, and VD_3_-treated PCOS group (*n* = 8 per each group) (Figure 1). The dose of VD_3_ was chosen based on previous [34] and our preliminary studies. A daily treatment regime of 21 days included oral administration through the gavage of either 2% (*v*/*v*) dimethyl sulfoxide (DMSO; Sigma-Aldrich) in rapeseed oil (1 mL/kg body weight) in the control group or letrozole (1 mg/kg body weight) dissolved in 2% DMSO in rapeseed oil. The rats were weighed daily for 21 consecutive days of the experiment, and the estrous cyclicity was assessed. The animals were deeply anesthetized via the inhalation of 4% isoflurane in an enclosed vessel. Trunk blood was collected in a tube and centrifuged at 4000× *g* for 10 min at 4 °C, and the plasma was stored at −20 °C for further analyses. The uterus and ovary were dissected out, fixed in formalin or snap-frozen in liquid nitrogen and stored at −80 °C for RNA and protein isolation. To verify PCOS induction, after 21 days of treatment, plasma levels of steroids: T and E_2_ and metabolic parameters: glucose, triglycerides, total cholesterol, and HDL as well as ovarian and uterus histology were analyzed.

### 2.3. Ovarian and Uterus Histology

Paraplast-embedded ovaries and uteri were cut into 5 μm thick sections and mounted on 3′3′-aminopropyl-triethoxysaline-coated slides. After deparaffinization and rehydration, tissue sections were stained with hematoxylin QS and eosin Y. Next, the stained slides were dehydrated, mounted in DPX, and coverslipped. Digital images were collected using an Axio Scope A1 microscope with EC Epiplan-NEOFLUAR objectives equipped with a Nikon Eclipse Ni-U microscope using a Nikon Digital DS-Fi1-U3 camera (Nikon, Japan) and Niko NIS-ELEMENT Image Software.

### 2.4. ELISA

The commercially available ELISA kits were used to determine the plasma level of T and E_2_ (cat no. EIA-1559, EIA-2693, respectively, DRG Instruments GmbH, Marburg, Germany). The sensitivity of the assays was 0.083 ng/mL for T and 9.714 pg/mL for E_2_. The inter- and intra-experimental coefficients of variation were <9.94% and <4.16% for T or <9.39% and <6.81% for E_2_. 

The commercially available ELISA kits were used to determine the plasma level of RARRES2 and ADIPOQ (cat. no. ER0834, FineTest, Wuhan, China; cat. no. E-EL-R3012, Elabscience, Houston, TX, USA, respectively). The sensitivity of the assays was 18.75 pg/mL for RARRES2 and 0.94 ng/mL for ADIPOQ. The inter- and intra-experimental coefficients of variation were <10% and <8% for RARRES2 as well as <5% and <4.5% for ADIPOQ. All analyses were performed in duplicate. 

### 2.5. Colorimetric Assays

The commercially available colorimetric assays were used to determine the plasma level of glucose, triglycerides, total cholesterol, and HDL (cat. no G7521, T7532, C7510, and H7511, respectively; Pointe Scientific, Warsaw, Poland). The concentration of the low-density lipoprotein (LDL) fraction was calculated using the following formula: LDL = total cholesterol-HDL (triglycerides/5). Detection limits were 1 mg/dL for glucose, 5 mg/dL for triglycerides, 3 mg/dL for total cholesterol, and 3 mg/dL for HDL. The intra-assay and inter-assay coefficients of variation for glucose, triglycerides, total cholesterol, and HDL were 1.1% and 2.5%, 1.62% and 1.92%, 1.32% and 1.93%, and 4.7% and 5.1%, respectively. All analyses were performed in duplicate.

### 2.6. Real-Time PCR

Total RNA was extracted from the uterus using TRI Reagent solution according to the manufacturer’s protocol. The quality of RNAs was evaluated by measuring the A260/A280 ratio using a NanoDrop™Lite Spectrophotometer (Thermo Scientific). Total RNA (1 µg) was used as a template in cDNA synthesis with High-Capacity cDNA Reverse Transcription Kit. Real-time PCR analyses were performed using StepOne Real-Time PCR (Applied Biosystems, Norwalk, CT, USA). TaqMan Gene Expression Assays were used to quantify the mRNA expression of adipokines and their receptor genes (Table 1). Quantitative PCR was performed with 100 ng cDNA, 1 mL TaqMan Gene Expression primers, and 10 mL TaqMan PCR master mix (Applied Biosystems) in a final reaction volume of 20 mL. After a 2 min incubation at 50 °C, the thermal cycling conditions were 10 min at 95 °C, followed by 40 cycles of 15 s at 95 °C and 1 min at 60 °C to determine the cycle threshold number (*C*t) for quantitative measurement. The relative mRNA expression levels of adipokines and their receptor genes relative to GAPDH were determined using the 2^−Δ*C*t^ method [35]. Briefly, the cycle threshold (*C*t; defined as the cycle number at which the fluorescence exceeds the threshold level) was determined for each sample. The *C*t value of the reference gene, GAPDH, was subtracted from the *C*t value of the gene of interest (Δ*C*t) and the relative expression was presented as 2^−Δ*C*t^. These 2^−Δ*C*t^ values were used to calculate the statistical differences. The values are expressed as a relatively arbitrary unit. 

### 2.7. Western Blotting

Western blotting and quantification were performed as previously described [36]. In brief, tissue was homogenized twice in ice-cold lysis buffer, and then the lysates were cleared by centrifugation at 15,000× *g* for 15 min at 4 °C. The protein content was determined via a protein assay using bovine serum albumin (BSA) as a standard and then Western blots for RARRES2 and its receptors CCRL2, CMKLR1, GPR1, as well as ADIPOQ and its receptors ADIPOR1 and ADIPOR2 in the uterine were performed. Briefly, 30 μg of protein was separated by 4–20% SDS–PAGE transferred onto a PVDF membrane (BioRad Mini-Protean 3 apparatus, Bio-Rad Laboratories). The blots were blocked for 2 h with 5% *w/v* BSA and 0.1% *v/v* Tween 20 in 0.02 M Tris-buffered saline buffer (TBS). Next, the blots were incubated with primary antibodies for anti-RARRES2 (cat. no. ab103153, Abcam, Waltham, MA, USA), anti-CCRL2 (cat. no. ab88632, Abcam), and anti-CMKLR1 (cat. no. SAB4500334, Sigma-Aldrich) diluted at 1:500 as well as anti-GPR1 (cat. no. ab169331, Abcam), anti-ADIPOQ (cat. no. PA1-054, Thermo Scientific), anti-ADIPOR1 (cat. no. PA5-35347, Thermo Scientific), and anti-ADIPOR2 (cat. no. PA1-12759, Thermo Scientific) diluted at 1:1000 at 4 °C overnight. Then, the membrane was incubated with secondary antibody anti-mouse (cat. no. 7076S, Cell Signaling, Beverly, MA, USA) or anti-rabbit (cat. no. 7074S, Cell Signaling) diluted at 1:1000 for 1 h before chemiluminescence using WesternBright Quantum HRP substrate and visualized using a Chemidoc XRS + System (Bio-Rad). All visible bands were quantified using a densitometer and ImageJ software (US National Institutes of Health, Bethesda, MD, USA). β-actin (ACTB) (cat. no. A5316, Sigma-Aldrich) was also detected as a loading control. Semi-quantitative analysis was performed for three separately repeated experiments.

### 2.8. Immunohistochemistry

Immunohistochemistry was conducted on formalin-fixed, paraffin-embedded, 5 μm thick uterine sections as previously described from four rats per each group [12]. Sections were deparaffinized in xylene, rehydrated in decreasing gradient of ethanol, and subjected to microwave (750 W) antigen retrieval in 0.01 M (*w*/*v*) citrate buffer (pH 6.0). Endogenous peroxidase activity was blocked with 0.3% (*v*/*v*) hydrogen peroxide in TBS for 20 min, and non-specific binding sites were blocked with 5% (*v*/*v*) normal goat (for ADIPOQ, ADIPOR1, and CMKLR1) or horse (for RARRES2) serum in TBS for 40 min. After overnight incubation at 4 °C in a humidified chamber with the primary antibodies anti-ADIPOQ (dilution 1:50), anti-ADIPOR1 (dilution 1:50), anti-RARRES2 (dilution 1:100), and anti-CMKLR1 (dilution 1:200), the sections were immersed for 1.5 h at room temperature with anti-rabbit or anti-mouse secondary antibodies (dilution 1:300; Vector Laboratories, USA) followed by the incubation with an avidin–biotin–peroxidase complex (dilution 1:100, 40 min at room temperature, StreptABComplex-HRP) and then with 3,3′-diaminobenzidine as a chromogen. Sections were then counterstained with hematoxylin QS, dehydrated, fixed in xylene, and mounted using DPX. Negative controls were incubated with non-immune rabbit or mouse IgGs instead of primary antibodies and processed as above. Selected sections were photographed under a Nikon Eclipse Ni-U microscope using a Nikon Digital DS-Fi1-U3 camera (Nikon, Tokyo, Japan) and Nikon NIS-ELEMENT Image Software. To evaluate the intensity of the immunoreaction quantitatively, digital images from at least 10 different sections of each examined animal were analyzed using ImageJ software. The intensity of RARRES2, CMKLR1, ADIPOQ, and ADIPOR1 staining was expressed as a relative optical density (ROD) and was calculated using the following formula: ROD = OD_specimen_/OD_background_ = log(GL_blank_/GL_specimen_)/log(GL_blank_/GL_background_); where *OD*—optical density, *GL*—grey level, *specimen*—stained area, *background*—unstained area, and *blank*—grey level measured after removing the slide from the light path.

### 2.9. Statistical Analysis

Statistical data are presented as the means ± standard error of the mean (SEM). The distribution of normality was checked with the Shapiro–Wilk test. Statistical analysis was carried out using one-way or two-way ANOVA, followed by Tukey’s test (PRISM software version 8; GraphPad, La Jolla, CA, USA). Statistically significant differences (*p* < 0.05) between points in each graph are indicated by different letters (a < b < c < d), identical letters indicate a lack of significant differences.

## 3. Results

### 3.1. Plasma Levels of Hormonal and Metabolic Parameters, and Ovarian Morphology in PCOS Rats

Plasma concentrations of steroid hormones and metabolic parameters are presented in Table 2. In PCOS rats, the plasma level of T increased, while the E_2_ concentration was lower compared to the control rats (*p* < 0.05). We noted elevated plasma levels of glucose, triglyceride, cholesterol, HDL, and LDL in PCOS rats vs. control (*p* < 0.05). 

**Figure 2:**
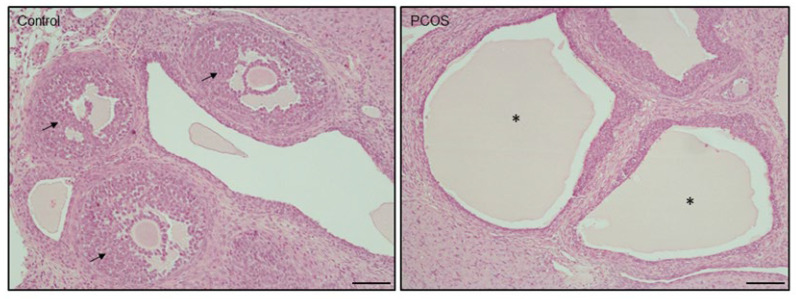
Representative photomicrographs of ovary from control and induced PCOS rats. Arrows indicate early antral follicles, while asterisks indicate ovarian cysts. Scale bar 100 µm. PCOS, polycystic ovarian syndrome.

We also observed that control rats displayed normal follicular development with preantral follicles and antral follicles, whereas PCOS rats resulted in ovarian cysts and a lack of corpora lutea (Figure 2).

### 3.2. Uterine Histology

**Figure 3:**
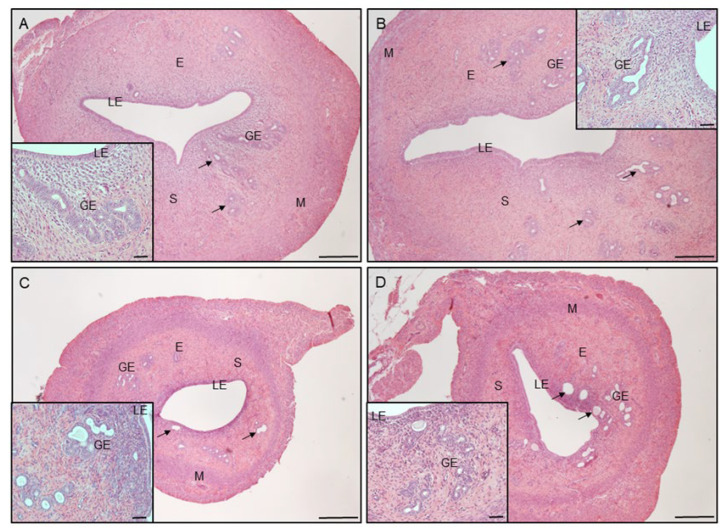
Hematoxylin and eosin staining of uterus obtained from control (**A**), VD_3_-treated (**B**), induced PCOS (**C**), and VD_3_-treated PCOS (**D**) rats. Scale bar 250 µm or 50 µm for insets. E, endometrium. M, myometrium. GE, glandular epithelium. LE, luminal epithelium. PCOS, polycystic ovarian syndrome. S, stroma. VD_3_, vitamin D_3_. Arrows indicate endometrial glands.

In all examined groups, uteri comprised the endometrium and the myometrium layers. The endometrium includes luminal (LE) and glandular epithelium (GE), and stroma cells (Figure 3A–D). However, in PCOS rats (Figure 3C) and in PCOS rats treated with VD_3_ (Figure 3D), the uteri were smaller in cross-sections, and the endometrial compartment was thinner in those groups (Figure 3C,D). The endometrial glands in PCOS (Figure 3C, arrows; inset) and PCOS rats treated with VD_3_ (Figure 3D, arrows; inset) were round and unbranched in comparison to the control (Figure 3A, arrows; inset) and VD_3_-treated (Figure 3B, arrows; inset) groups.

### 3.3. Effect of VD_3_ on Plasma Level of RARRES2, ADIPOQ and Steroids in PCOS Rats

**Figure 4:**
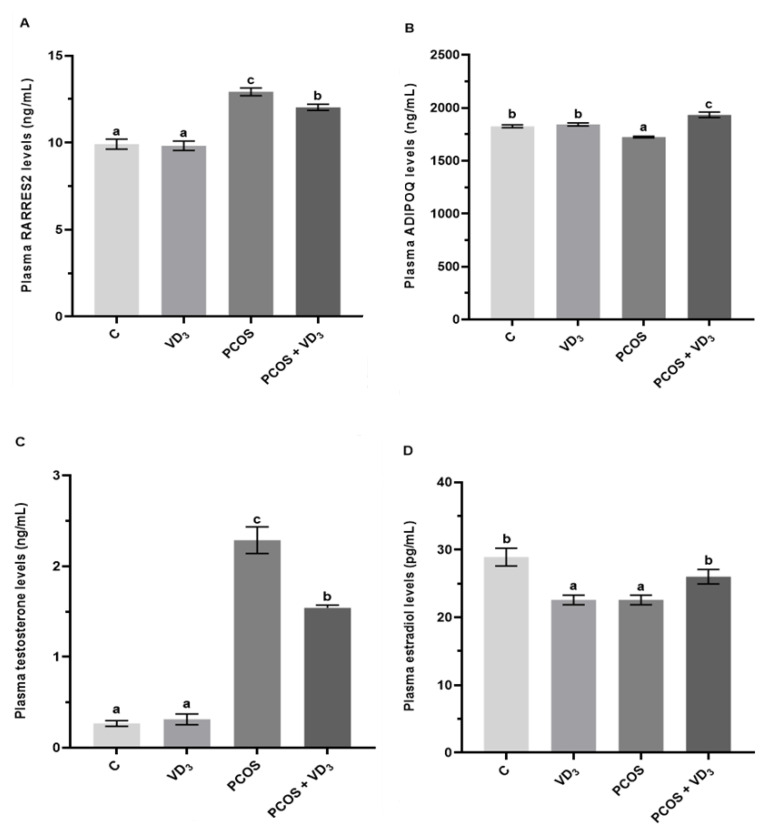
Plasma concentrations of RARRES2 (**A**), ADIPOQ (**B**), testosterone (**C**), and estradiol (**D**) measured by ELISA assay. Results were mean ± SEM of eight independent determinations. Statistically significant differences (*p* < 0.05) between points in each graph are indicated by different letters (a < b < c), identical letters indicate a lack of significant differences. ADIPOQ, adiponectin. C, control. ELISA, enzyme-linked immunosorbent assay. PCOS, polycystic ovarian syndrome. RARRES2, retinoic acid 109 receptor responder protein 2. SEM, standard error of the mean. VD_3_, vitamin D_3_.

As shown in Figure 4A, VD_3_ treatment did not affect RARRES2 plasma concentration in control rats, while in PCOS rats, we noted higher levels of RARRES2 (*p* < 0.05). In PCOS rats supplemented with VD_3_, we found decreased plasma levels of RARRES2 compared to the PCOS group but not to the control levels (Figure 4A, *p* < 0.05). The plasma concentration of ADIPOQ in the VD_3_-treated group was not changed but was decreased in PCOS rats (Figure 4B, *p* < 0.05). VD_3_ supplementation in PCOS rats resulted in an increase in ADIPOQ levels toward control values.

We observed that the plasma level of T did not change in the VD_3_-treated control group, increased in PCOS rats, but markedly decreased in PCOS supplemented with VD_3_ vs. PCOS rats, but not vs. the control levels (Figure 4C, *p* < 0.05). The plasma E_2_ concentration decreased in VD_3_-treated and PCOS rats vs. control; however, in PCOS rats supplemented with VD_3_, E_2_ increased to the same level as in the control group (Figure 4D, *p* < 0.05).

### 3.4. Effect of VD_3_ on Gene and Protein Expression of RARRES2 and Its Receptors in the Uterus of PCOS Rats

**Figure 5:**
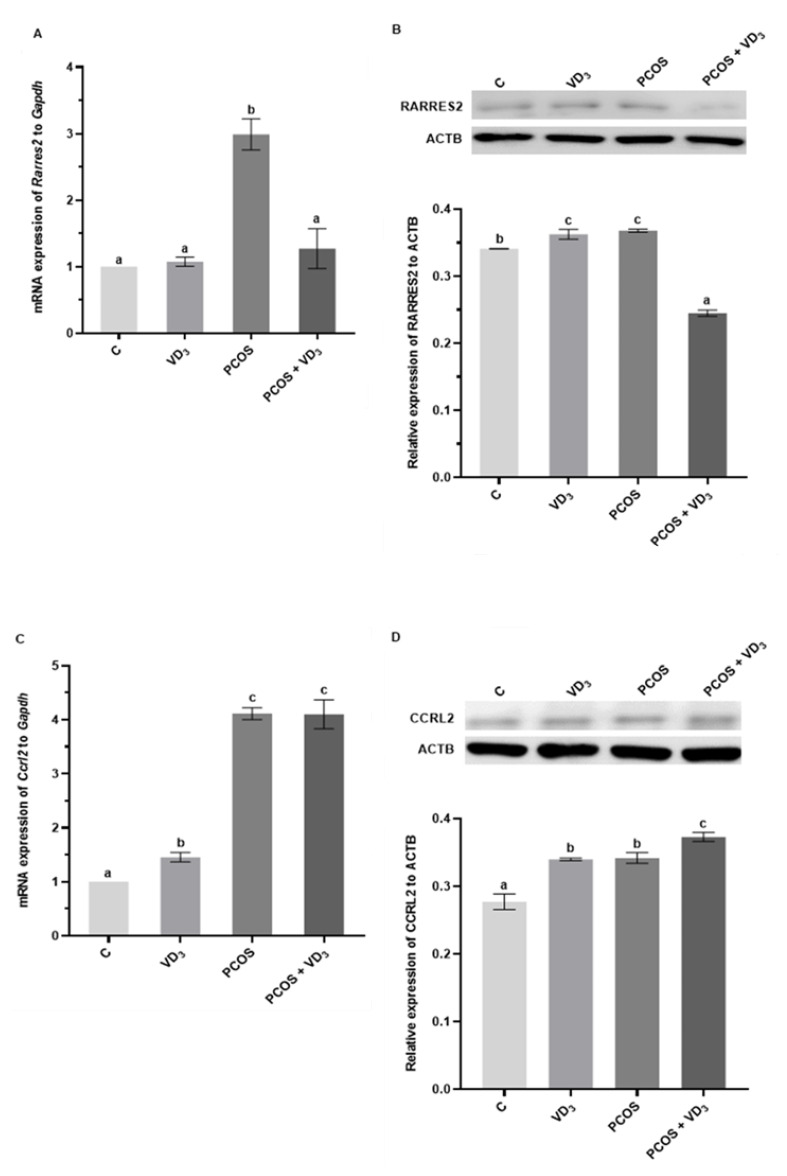

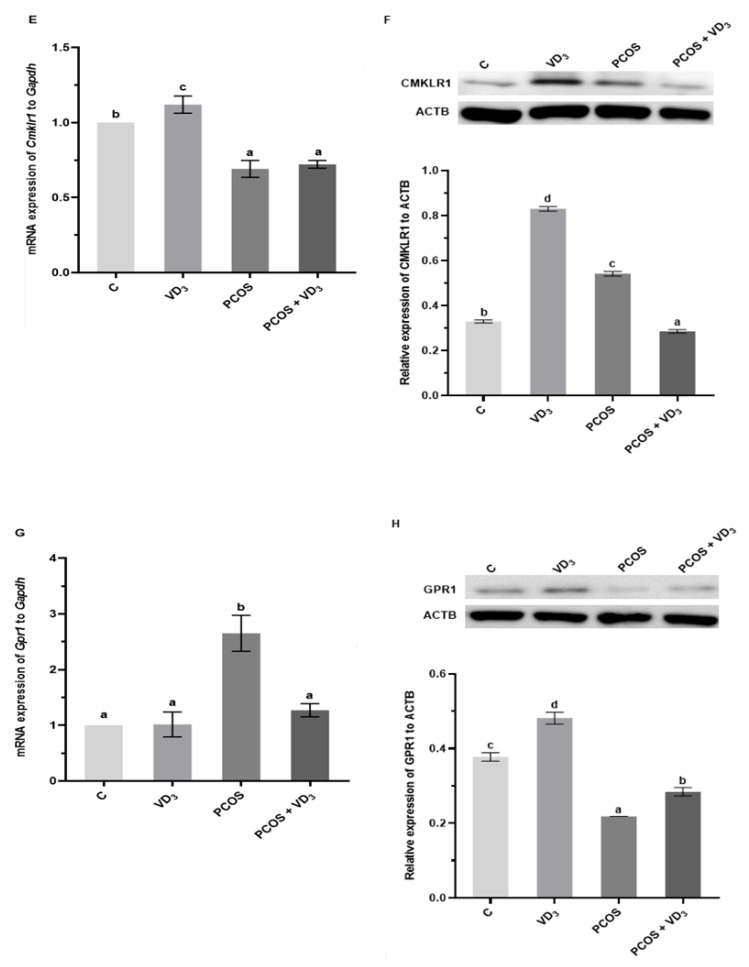
mRNA and protein expression of RARRES2 (**A**,**B**), CCRL2 (**C**,**D**), CMKLR1 (**E**,**F**), and GPR1 (**G**,**H**) in rat uterus. mRNA expression was determined using real-time PCR and presented relative to *Gapdh*; the protein expression was detected via Western blot assay, each protein abundance was evaluated densitometrically and expressed as the ratio relative to the ACTB abundance. Results were mean ± SEM of eight independent determinations. Statistically significant differences (*p* < 0.05) between points in each graph are indicated by different letters (a < b < c < d), identical letters indicate a lack of significant differences. ACTB, β-actin. C, control. CCRL2, C-C motif chemokine receptor-like 2. CMLR1, chemerin chemokine-like receptor 1. *Gapdh*, glyceraldehyde-3-phosphate dehydrogenase. GPR1, G protein-coupled receptor 1. PCR, polymerase chain reaction. PCOS, polycystic ovarian syndrome. RARRES2, retinoic acid 109 receptor responder protein 2. SEM, standard error of the mean. VD_3_, vitamin D_3_.

The gene expression of *Rarres2* was unchanged in the uterus of the VD_3_-treated control group, whereas was higher in PCOS rats (Figure 5A, *p* < 0.05), and decreased to the control level in PCOS rats treated with VD_3_. However, we noted that the protein expression of RARRES2 was greater in the uterus of VD_3_-treated and PCOS rats, while in PCOS supplemented with VD_3_, we observed a lower expression of RARRES2 in the uterus vs. the control group (Figure 5B, *p* < 0.05).

We demonstrated that *Ccrl2* mRNA expression markedly increased in the uterus of all experimental groups: VD_3_-treated, PCOS-induced, and PCOS-supplemented with VD_3_ (Figure 5C, *p* < 0.05). These results were confirmed for the protein levels of CCRL2 in the uterus (Figure 5D, *p* < 0.05).

The transcript levels of *Cmklr1* in the uterus were higher in the VD_3_-treated group vs. the control group (Figure 5E, *p* < 0.05). We observed that in the uterus of PCOS rats, the expression of *Cmklr1* was lower compared to the control group, and the supplementation of PCOS rats with VD_3_ did not differ between PCOS rats but decreased vs. the control. The protein abundance of CMKLR1 in the uterus was higher in the VD_3_-treated and PCOS groups (Figure 5F, *p*  <  0.05) when compared to the controls, while in PCOS rats treated with VD_3_, we noted lower CMKLR1 expression vs. the control. 

The gene expression of *Gpr1* was higher in the uterus in the PCOS group compared to the control rats (Figure 5G, *p* < 0.05). The supplementation of VD_3_ in the PCOS rats decreased transcript *Gpr1* expression in the uterus to the control level. However, the protein expression of GPR1 markedly increased in the uterus of the VD_3_-treated group, while in the PCOS and PCOS treated with VD_3_ groups, it was significantly decreased (Figure 5H, *p* < 0.05).

### 3.5. Effect of VD_3_ on Cellular Immunolocalization of RARRES2 and CKMLR1 in the Uterus of PCOS Rats

**Figure 6:**
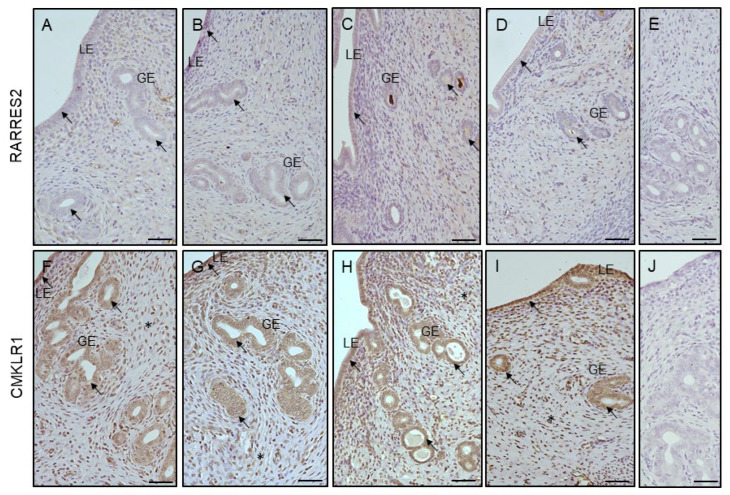

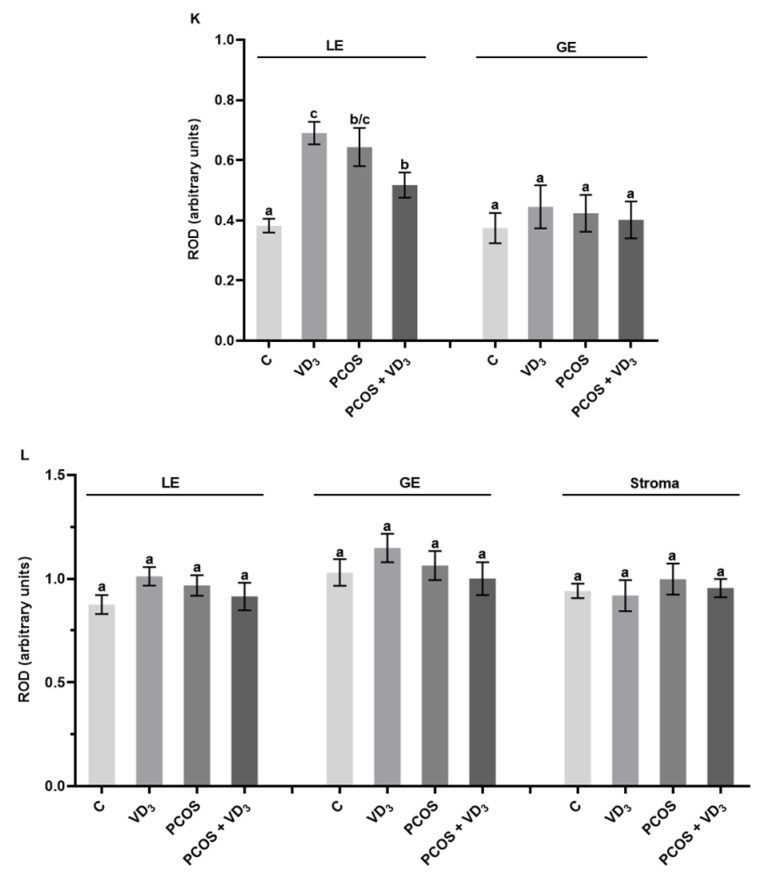
Representative micrographs of immunohistochemical RARRES2 and CMKLR1 localization in uterus obtained from control ((**A**) and (**F**), respectively), VD_3_-treated ((**B**) and (**G**), respectively), induced PCOS ((**C**) and (**H**), respectively), and VD_3_-treated PCOS ((**D**) and (**I**), respectively) rats. Arrows indicate positive RARRES2 and CMKLR1 staining within LE and GE in all examined groups ((**A**–**D**) and (**F**–**I**), respectively). Asterisks indicate positive CMKLR1 immunoreaction in stroma cells (**F**–**I**). Control sections ((**E**) and (**J**), respectively) showed no positive staining. Charts represent the intensity of RARRES2 (**K**) and CMKLR1 (**L**) immunostaining expressed as the relative optical density (ROD) in LE, GE, and stroma. Statistically significant differences (*p* < 0.05) between points in each graph are indicated by different letters (a < b < c), identical letters indicate a lack of significant differences. Scale bar 50 μm. C, control. CMKLR1, chemerin chemokine-like receptor 1. GE, glandular epithelium. LE, luminal epithelium. RARRES2, retinoic acid 109 receptor responder protein 2. PCOS, polycystic ovarian syndrome. SEM, standard error of the mean. VD_3_, vitamin D_3_.

RARRES2 and CMKLR1 were found in LE and GE, while CMKLR1 appeared additionally in the stroma cells of all examined groups (Figure 6). The intensity of RARRES2 immunoreaction within LE was greater in the VD_3_-treated, PCOS, and PCOS-and-VD_3_-treated groups than in the control, and lower in the PCOS-and-VD_3_-treated rats than in the VD_3_-treated rats (Figure 6K, *p* < 0.05). The intensity of the RARRES2 immunoreaction within GE did not differ between the groups (Figure 6K). Regarding the intensity of the CMKLR1 immunoreaction, there were no significant differences between the examined groups either in LE or GE and stroma (Figure 6L).

### 3.6. Effect of VD_3_ on Gene and Protein Expression of ADIPOQ and Its Receptors in the Uterus of PCOS Rats

**Figure 7:**
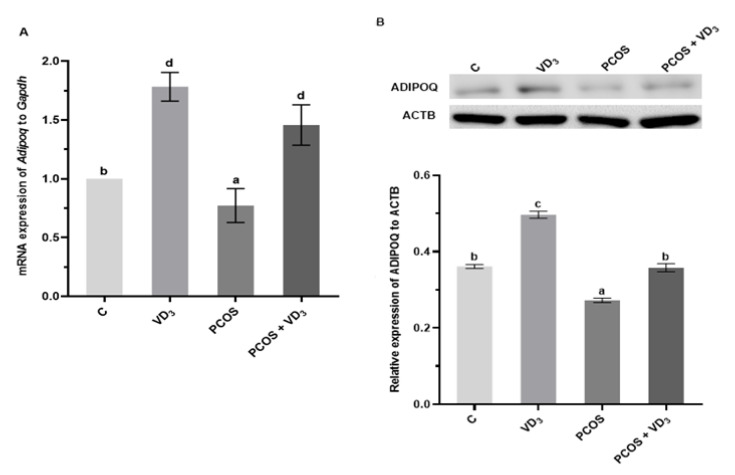

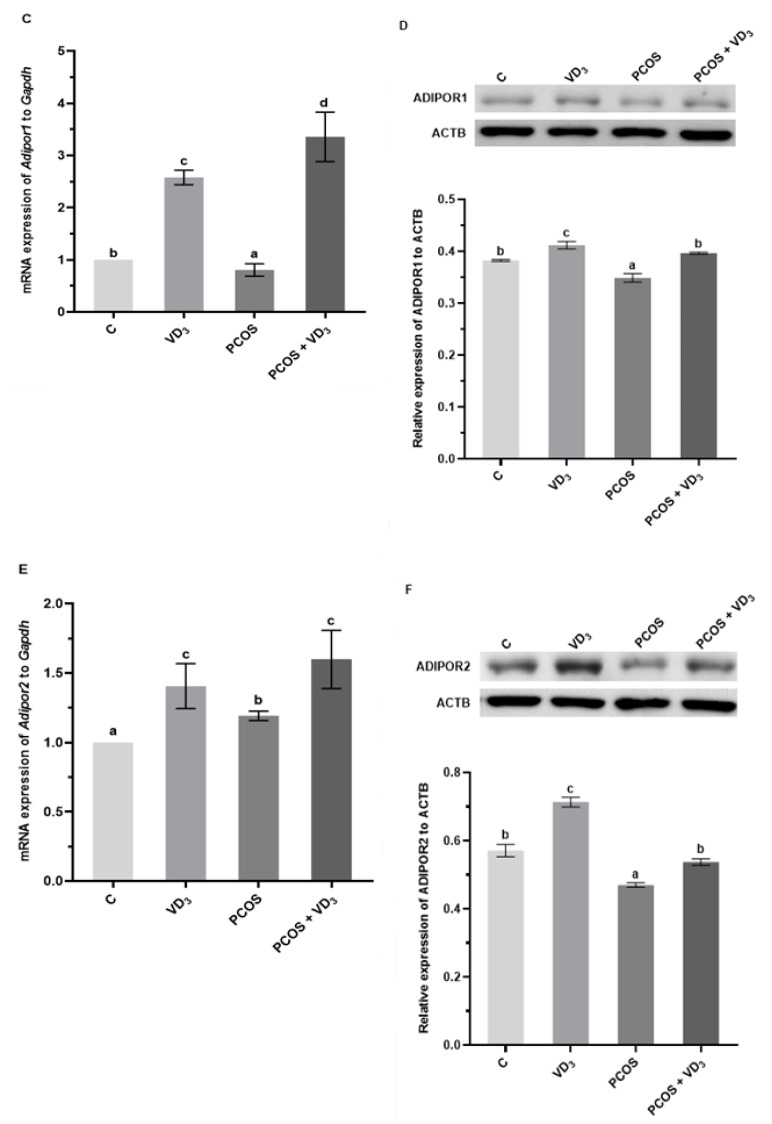
mRNA and protein expression of ADIPOQ (**A**,**B**), ADIPOR1 (**C**,**D**), and ADIPOR2 (**E**,**F**) in rat uterus. mRNA expression was determined using real-time PCR and presented relative to *Gapdh*; the protein expression was detected by Western blot assay; each protein abundance was evaluated densitometrically and expressed as the ratio relative to ACTB abundance. Results were mean ± SEM of six independent determinations. Statistically significant differences (*p* < 0.05) between points in each graph are indicated by different letters (a < b < c < d), identical letters indicate a lack of significant differences. ACTB, β-actin. ADIPOQ, adiponectin. ADIPOR1, adiponectin receptor-1. ADIPOR2, adiponectin receptor-2. C, control. *Gapdh*, glyceraldehyde-3-phosphate dehydrogenase. PCR, polymerase chain reaction. PCOS, polycystic ovarian syndrome. SEM, standard error of the mean. VD_3_, vitamin D_3_.

As shown in Figure 7A, the mRNA expression of *Adipoq* in the uterus of the VD_3_-treated group was higher, while in the PCOS group, it was decreased compared to the control rats (*p* < 0.05). We observed that in the PCOS rats supplemented with VD_3_, the expression of *Adipoq* increased vs. the control group. A similar effect was noted in the protein abundance of ADIPOQ in the uterus of all experimental groups, except PCOS rats treated with VD_3_, where the ADIPORQ was at the same level as in the control rats (Figure 7B, *p* < 0.05).

The expression of *Adipor1* mRNA in the uterus was higher in the VD_3_-treated vs. the control group, lower in PCOS rats, and increased in the PCOS supplemented with VD_3_ groups (Figure 7E, *p* < 0.05). A similar effect we found at the protein levels in the uterus of ADIPOR1 in VD_3_-treated and PCOS rats, while in the PCOS-treated VD_3_ group, the ADIPOR1 protein expression increased to the control level (Figure 7D, *p* < 0.05).

We noted that transcript levels of *Adipor2* increased in the uterus of all experimental groups (Figure 7E, *p* < 0.05). For the protein level, we observed that ADIPOR2 in the uterus markedly increased in the VD_3_ and decreased in the PCOS groups (Figure 7D, *p* < 0.05), while in the PCOS rats supplemented with VD_3_, the protein expression of ADIPOR1 increased to the control level.

### 3.7. Effect of VD_3_ on Cellular Immunolocalization of ADIPOQ and AdipoR1 in the Uterus of PCOS Rats

**Figure 8:**
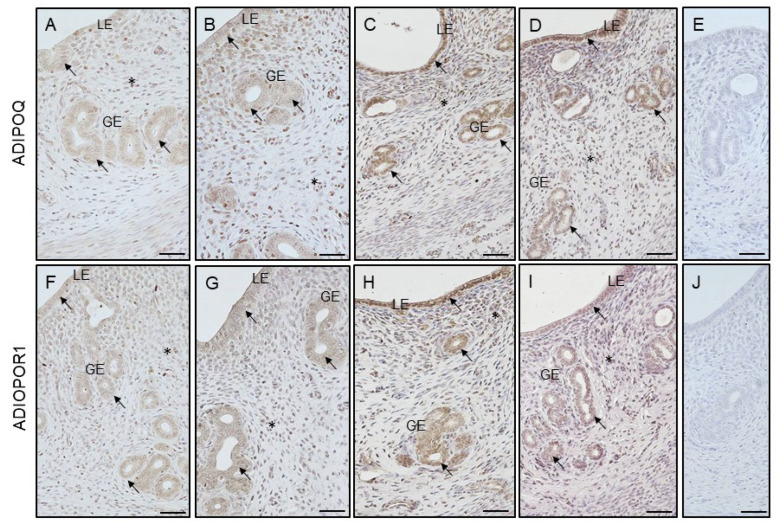

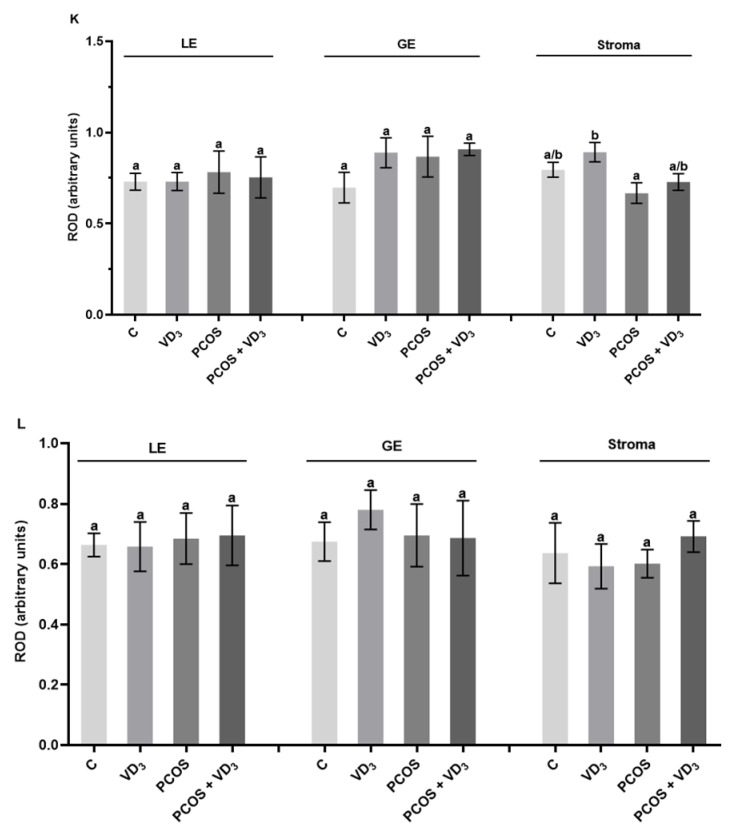
Representative micrographs of immunohistochemical ADIPOQ and ADIPOR1 localization in uterus obtained from control ((**A**) and (**F**), respectively), VD_3_-treated ((**B**) and (**G**), respectively), induced PCOS ((**C**) and (**H**), respectively) and VD_3_-treated PCOS ((**D**) and (**I**), respectively) rats. Arrows indicate positive ADIPOQ and ADIPOR1 staining within LE and GE ((**A**–**D**) and (**F**–**I**), respectively), but asterisks in stroma cells ((**A**–**D**) and (**F**–**I**), respectively) of all examined groups. Control sections ((**E**) and (**J**), respectively) showed no positive staining. Charts represent the intensity of ADIPOQ (**K**) and ADIPOQR1 (**L**) immunostaining expressed as the ROD in LE, GE, and stroma. Statistically significant differences (*p* < 0.05) between points in each graph are indicated by different letters (a < b), identical letters indicate a lack of significant differences. Scale bar 50 μm. ADIPOQ, adiponectin. ADIPOR1, adiponectin receptor-1. GE, glandular epithelium. LE, luminal epithelium. PCOS, polycystic ovarian syndrome. ROD, relative optical density. SEM, standard error of the mean. VD_3_, vitamin D_3_.

ADIPOQ and ADIPOR1 were detected in the LE and GE as well as in the stromal cells of the control (Figure 8A,F, respectively), VD_3_ (Figure 8B,G, respectively), PCOS (Figure 8C,H, respectively), and PCOS treated with VD_3_ (Figure 8D,I, respectively) groups. The intensity of the ADIPOQ immunoreaction was similar between the groups within the LE and GE, while in the stroma, the ADIPOQ signal was lower in the PCOS rats than in the VD_3_-treated animals (Figure 8K, *p* < 0.05). The intensity of the ADIPOR1 immunoreaction did not differ between the groups within the LE, GE, and stroma (Figure 8L).

## 4. Discussion

A positive effect of VD_3_ supplementation on the uterus alteration of PCOS patients was demonstrated in previous studies. For example, VD_3_ treatment could increase endometrial thickness in intrauterine-inseminated women with PCOS and increase the success rate of intrauterine insemination [37]. Moreover, supplementation with calcium and ergocalciferol for 2 months improved the menstrual regularity of women with PCOS and hypovitaminosis D [38]. A pilot study revealed that supplementation with VD_3_ improves glucose metabolism and menstrual frequency in PCOS women [39] and gonadotropin concentrations in induced PCOS in rats [40]. Our previous study demonstrates that the ovarian conversion of the circulating 25OHD_3_ to active 1,25(OH)_2_D_3_ is decreased in the PCOS rat model, and this may contribute to the VD_3_ deficiency observed in PCOS [12]. To the best of our knowledge, this is the first study investigating the effect of VD_3_ supplementation in PCOS rats on plasma levels of two adipokines—RARRES2 and ADIPOQ—as well as the transcript and protein expression of these adipokines and their receptors in the uterus. We found that VD_3_ supplementation does not influence the plasma levels of RARRES2 and ADIPOQ, while in PCOS rats supplemented with VD_3_, the concentration of plasma adipokines returns to the levels of the control group. Moreover, we noted variations in the gene and protein expression of RARRES2 and ADIPOQ and their receptors in the uterus of PCOS rats. In our study, we used a letrozole-induced rat model of PCOS, which exhibits hormonal, reproductive, and metabolic signs similar to PCOS women [33]. In the PCOS rats, we observed significantly higher plasma concentrations of T and lower E_2_ levels compared to the control. Additionally, metabolic parameters like glucose, triglyceride, cholesterol, HDL, and LDL were increased in the PCOS rats. Moreover, in the PCOS ovaries, we noted cystic follicles and a lack of corpora lutea; therefore, the PCOS model was confirmed via histology, endocrinology, and metabolic parameters in the present study, as previously described [41]. The metabolic dysfunction of PCOS women also contributed to increased endometrial thickness and hyperplasia [42]. 

Treatment with VD_3_ in rats with PCOS significantly decreased the T concentration and increased the E_2_ levels. Our study, in agreement with Behmanesh et al. [40], demonstrated significant PCOS-related changes in steroids: P_4_, T, and E_2_ levels. Moreover, VD_3_ supplementation in PCOS rats increased insulin sensitivity and thereby stimulated the development of the dominant follicles and the ovulation of matured follicles, suggesting that VD_3_ treatment can protect ovarian tissue from the negative effects of PCOS [40]. Besides the ovary, hormonal changes also influence the uterus histology. We have observed that the uterus in PCOS and PCOS treated with VD_3_ rats was smaller in cross-section with thinner endometrium and unbranched glands in comparison to the control and VD_3_-treated groups. That is in agreement with the results of Yan et al. [7], who noted reduced uterine weight, endometrium thickness, and number of endometrial glands following letrozole administration. 

It has been reported that the plasma levels of circulating adipokines are changed in PCOS patients [43] and they are a link between infertility related to obesity and the development of PCOS. ADIPOQ is the most abundantly secreted adipokine by the adipose tissue, and the latest data show that there is a significantly lower circulating level of ADIPOQ in PCOS women [43]. On the other hand, PCOS women have elevated levels of RARRES2, which is associated with insulin resistance and inflammation [44]. In our study, we observed an elevated plasma level of RARRES2 in PCOS rats compared to the control group, which is in agreement with the results obtained in PCOS women [43]. The serum concentration of RARRES2 was positively correlated with the T level in women [45] and negatively correlated with the serum E_2_ level in men [46], which we also observed in induced PCOS rats. Interestingly, our data show that VD_3_ treatment has no effect on plasma RARRES2 levels; however, we observed that after VD_3_ supplementation in PCOS rats, the concentration of RARRES2 was significantly reduced to the control level. Literature data on the effect of VD_3_ on RARRES2 levels are limited. Nassar and Badae [17] observed that in a rat model of PE supplementation of VD_3_ decreased serum RARRES2 levels, which was significantly increased in PE-untreated rats. Additionally, Abeer et al. [47], demonstrated that VD_3_ supplementation in gestational-diabetes-mellitus-induced rats reduces circulating RARRES2 levels, which partly confirms the results obtained in the present study. Our results showed that the transcript and protein levels of RARRES2 and its receptors, CCRL2, CMKLR1, and GPR1, were markedly changed in the uterus after VD_3_ supplementation. We observed that levels of RARRES2 and all its receptors increased in the uterus in VD_3_-treated rats, except for *Rarres2* and *Gpr1* mRNA expression. Interestingly, VD_3_ supplementation in PCOS rats decreased the uterine expression of RARRES2 and the receptors CMKLR1 and GPR1 but increased CCRL2. In our data, we observed a difference in the transcripts’ contents and the corresponding abundance of proteins. Schwanhäusser et al. [48] suggest that such differences in the transcripts and the corresponding proteins could arise from mRNA binding by specific proteins, the regulation of expression by microRNAs, and RNA and protein stability. Similarly, our previous study documented that the *Smim20* (precursor of neuropeptide phoenixin-14) mRNA expression in the hypothalamus of PCOS rats was not parallel with the phoenixin-14 protein levels [41]. Moreover, the difference in the transcript and protein levels of RARRES2 and its receptors can be explained by transcriptional, post-transcriptional, and translational regulations and discrepancies in mRNA and protein stability [49]. RARRES2 and CMKLR1 were found in the luminal and glandular epithelium, while CMKLR1 was additionally found in the stroma cells of all examined groups. In addition, the intensity of the RARRES2 immunoreaction was changed within the luminal epithelium and was lower in PCOS rats treated with VD_3_ than in the group supplemented with VD_3_ alone, which is partly similar to results obtained herein from Western blot analysis. Our results are partly in agreement with the data of Roman and Sinal [50], who observed that VD_3_ may regulate the expression of RARRES2 in human adipocyte precursors. On the other hand, Nagpal et al. [51] demonstrated that VD_3_ has no effect on the total RNA level of *RARRES2* in human skin raft cultures, which we also observed in the uterus. The results of this study indicated that only the expression of CCRL2 increased significantly in the uterus of PCOS rats supplemented with VD_3_. The data of De Henau et al. [52] demonstrated that, unlike CMKLR1 and GPR1, CCRL2 does not mediate the signal transduction/intracellular signaling pathway and is presently considered as an atypical receptor able to present the protein to cells expressing CMKLR1, so the molecular mechanism of our observation should be explained in future studies. CCRL2 is expressed by many cell types including leukocyte subsets and endothelial cells, and its expression is strongly upregulated by inflammatory stimuli [53]. Additionally, previous data demonstrated that RARRES2 regulates P_4_ and E_2_ synthesis [54] as well as affects the transcriptomic profile of the porcine endometrium, controlling the expression of numerous genes, including those involved in the cell migration and adhesion, angiogenesis, inflammation, and steroidogenesis [27]. Our study for the first time documented that levels of RARRES2 and all receptors, except the *Cmklr1* transcript and GPR1 protein, increased in the uterus of PCOS rats. This is in line with the results of Luo et al. [55], who observed that the expression of RARRES2 and CMKLR1 was higher in the ovary of the rat model of PCOS. Additionally, previous reports documented that RARRES2, through CMKLR1, regulates steroid secretion in granulosa cells collected from PCOS women [56]. Our study provides novel data on PCOS endocrinology; the supplementation of VD_3_ in PCOS rats decreased the plasma level of RARRES2 and the uterine expression of RARRES2, CMKLR1, and GPR1.

In the present study, we also demonstrated that the plasma ADIPOQ level did not change after VD_3_ treatment, decreased in PCOS rats, and increased in VD_3_-supplemented PCOS rats. Literature data suggest that in humans, the plasma ADIPOQ concentration negatively correlates with body mass index and insulin resistance and is lower in patients with type 2 diabetes [57] and PCOS [43]. Meta-analysis data showed no significant effect of VD_3_ on the plasma concentration of ADIPOQ [58], which is in line with our current results. However, we found that in VD_3_-treated rats, the transcript and protein levels of ADIPOQ and both receptors, ADIPOR1 and ADIPOR2, significantly increased in the uterus. Our results are in accordance with data from previous studies which indicated that VD_3_ treatment elevates the protein expression of ADIPOQ in adipose cells via post-transcription-dependent mechanisms involving endoplasmic reticulum proteins [59]. On the other hand, Lorente-Cebrián et al. [60] documented that VD_3_ did not affect ADIPOQ mRNA expression in human subcutaneous adipose tissue while decreasing ADIPOQ secretion. Little is known about the molecular mechanism of VD_3_’s impact on ADIPOQ levels. Literature data suggest that VD_3_ may affect ADIPOQ levels through the renin–angiotensinogen system by decreasing angiotensin production; this biomolecule is associated with the production of dysfunctional adipocytes, and, in consequence, it may lead to decreased ADIPOQ production [61]. Other studies suggest that VD_3_ may be associated with increased serum ADIPOQ levels by decreasing the gene expression of TNFα [62]. Additionally, other factors like how the peroxisome proliferator-activated receptor gamma (PPAR-γ) agonist increases the expression of ADIPOR2, and insulin inhibits its expression through the phosphoinositide 3-kinases pathway in porcine preadipocytes [63]. In the present study, we observed that VD_3_ supplementation in PCOS rats increased the transcript abundance of *Adipoq*, *Adipor1*, and *Adipor2* in the uterus and led to the return of the protein levels to the control values. Our results are in agreement with Seyyed et al. [19], who observed that in PCOS women treated with VD_3_, the ADIPOQ plasma level was significantly increased. Comim et al. [64] show that in antral follicles, *Adipor1* and *Adipor2* mRNA expression was reduced in theca cells from polycystic ovaries compared with theca from normal ovaries, while no differences were observed between polycystic ovaries and normal ovaries in the proportions of granulosa cells in antral follicles expressing ADIPOR1 or ADIPOR2. Moreover, silencing ADIPOR1 and ADIPOR2 in theca cells results in the enhanced production of androgens [64]. ADIPOQ and ADIPOR1 were detected in the luminal and glandular epithelium as well as in the stromal cells of the control, VD_3_, PCOS, and PCOS supplemented with VD_3_ groups. However, there were no significant differences in the intensity of the ADIPOQ and ADIPOR1 immunoreaction between the analyzed groups within the luminal and glandular epithelium as well as in the stroma cells.

## 5. Conclusions

In conclusion, to the best of our knowledge, the present study is the first to document the effect of VD_3_ supplementation on the plasma levels of RARRES2 and ADIPOQ, as well as their transcript and protein expression in the uterus of PCOS rats. VD_3_ supplementation in PCOS rats decreased the expression of RARRES2, CMKLR1, and GPR1 but increased the CCRL2 levels. Moreover, VD_3_ supplementation in PCOS rats increased the transcript of *Adipoq*, *Adipor1*, and *Adipor2* in the uterus and brought them to the control protein expression. Our findings indicate a new mechanism of action of VD_3_ in the uterus physiology in PCOS rats. 

## Figures and Tables

**Table 1:** TaqMan gene expression assays were used to quantify the mRNA expression for adipokines and their receptors.

Gene Symbol	Gene Name	Catalog Number	Reference Sequence
*Rarres2*	retinoic acid 109 receptor responder protein 2	Rn01451853_m1	NM_001013427.1
*Ccrl2*	chemokine (C-C motif) receptor-like 2	Rn01746782_g1	NM_001108191.1
*Cmklr1*	chemokine-like receptor 1	Rn00573616_s1	NM_022218.2
*Gpr1*	G protein-coupled receptor 1	Rn00564179_s1	NM_012961.1
*Adipoq*	adiponectin	Rn00595250_m1	NM_144744
*Adipor1*	adiponectin receptor-1	Rn01483784_m1	NM_207587.1
*Adipor2*	adiponectin receptor-2	Rn01463173_m1	NM_001037979.1
*Gapdh*	Glyceraldehyde-3-phosphate dehydrogenase	Rn01775763_g1	NM_017008.4

**Table 2:** Plasma levels of hormones and metabolic parameters in control and PCOS rats. HDL, high-density lipoprotein. LDL, low-density lipoprotein. PCOS, polycystic ovarian syndrome. Statistically significant differences (*p* < 0.05) between control and PCOS rats are indicated by different letters (a < b).

Hormone and Metabolic Parameters	Control	PCOS Rats
Testosterone (ng/mL)	0.268 ^a^	2.471 ^b^
Estradiol (pg/mL)	28.918 ^b^	22.575 ^a^
Glucose (mg/dL)	186.685 ^a^	281.391 ^b^
Triglyceride (mg/dL)	59.490 ^a^	98.668 ^b^
HDL	26.303 ^a^	28.067 ^b^
LDL	3.950 ^a^	17.735 ^b^
Cholesterol (mg/dL)	42.615 ^a^	66.538 ^b^

## Data Availability

The comprehensive study protocol is available from the authors. Data can be provided by the authors upon request.
